# Theory of the Action of Iodide of Potassium

**Published:** 1877-07

**Authors:** 


### THEORY OF THE ACTION OF IODIDE OF POTASSIUM.

We learn from the *Practitioner* that Dr. Kammerer explains the medicinal action of this salt by referring to its decomposition by the action of ozonized oxygen and by carbonic acid gas. In solutions of potassium iodide, ozone causes separation of free iodine ; and when in dilute aqueous solution the pressure of carbonic acid gas breaks up the salt into hydrogen iodide (hydriodic acid) and potassium bicarbonate, but the free hydriodic acid is readily decomposed by free oxygen, even when not ozonized into iodine and water. Now iodide of potassium, when introduced into the stomach, is absorbed directly into the blood. Here it meets with a large quantity of carbonic acid gas at a high pressure, and is decomposed into hydriodic acid and potassium bicarbonate, and the former is then immediately split up by the oxygen in the blood, into free iodine and water. And even if the decomposition of the iodide by the carbonic acid does not take place, the oxygen in the blood, which closely resembles ozone in its properties, is capable of setting the iodine free. This free iodine does not act upon the inorganic constituents of the blood, since, on the one hand, the bicarbonates are not decomposed by it, and, on the other, between potassium phosphate and iodine a reaction may take place, leading to the formation of an inferior oxide of iodine (subiodic acid), which last is reducible with extreme facility, and consequently effects rapid combustion of organic material, free iodine being at the same time set free, which again becomes converted into hydridic acid, to undergo the same series of changes. The action of iodide of potassium in augmenting the temperature of the blood and causing emaciation, Dr. Kammerer considers to be fully explained by the action of the drug in increasing the combustion operations in the blood.—*Medical and Surgical Reporter.*

